# Construction of pseudomolecule sequences of *Brassica rapa* ssp. *pekinensis* inbred line CT001 and analysis of spontaneous mutations derived via sexual propagation

**DOI:** 10.1371/journal.pone.0222283

**Published:** 2019-09-09

**Authors:** Jee-Soo Park, Ji-Hyun Park, Young-Doo Park

**Affiliations:** Department of Horticultural Biotechnology, Kyung Hee University, Yongin, Korea; Clemson University, UNITED STATES

## Abstract

Chinese cabbage (*Brassica rapa* ssp. *pekinensis*) is a major crop that is widely cultivated, especially in Korea, Japan, and China. With the advent of next generation sequencing technology, the cost and time required for sequencing have decreased and the development of genome research accelerated. Genome sequencing of Chinese cabbage was completed in 2011 using the variety Chiifu-401-42, and since then the genome has been continuously updated. In the present study, we conducted whole-genome sequencing of Chinese cabbage inbred line CT001, a line widely used in traditional or molecular breeding, to improve the accuracy of genetic polymorphism analysis. The constructed CT001 pseudomolecule represented 85.4% (219.8 Mb) of the Chiifu reference genome, and a total of 38,567 gene models were annotated using RNA-Seq analysis. In addition, the spontaneous mutation rate of CT001 was estimated by resequencing DNA obtained from individual plants after sexual propagation for six generations to estimate the naturally occurring variations. The CT001 pseudomolecule constructed in this study will provide valuable resources for genomic studies on Chinese cabbage.

## Introduction

Brassicaceae is the fifth largest family of flowering plants, comprising 338 genera and approximately 3,700 species [1]. The family is present in various climatic regions and is cultivated worldwide. Brassicaceae crops, including cabbage (*Brassica oleracea*), rapeseed (*Brassica napus*), and Chinese cabbage (*Brassica rapa*), are economically important as they are major vegetable crops in Korea [2,3].

With the recent development of next generation sequencing (NGS) technology, it has become possible to produce a considerable volume of information in a short period of time at low cost [4,5]. In addition, advances in algorithms and technologies facilitated the development of genome research [6,7]. NGS technology thus helps to improve the speed and accuracy of molecular breeding.

The advancement of NGS facilitated the completion of draft genomes of various crops and consequently galvanized genomic research [8]. It has been estimated that there are presently about 370,000 plant species worldwide [9], and genome databases for about 200 species have been published to date [10,11]. *Arabidopsis thaliana*, which has a small genome of 125 Mbp [12], was the first plant to be sequenced, thus establishing the basis for gene discovery and understanding of genomes in other plants [13,14]. The generated plant genomes have provided insight into diversity and evolution by assisting the development of genomic analyses such as the discovery and analysis of high-quality single nucleotide polymorphisms (SNPs), development of genetic markers, genotyping by sequencing, and genome-wide association studies.

From among the Brassicaceae crops, *B*. *rapa* ssp. *pekinensis* ‘Chiifu-401-42’ has been selected for the *Brassica* A genome in the *B*. *rapa* Genome Sequencing Project (BrGSP). Multinational research groups from Korea, China, Japan, the United Kingdom, Canada, and the United States were involved in the BrGSP consortium to decode the genome sequence of *B*. *rapa* and develop suitable genomic resources [15]. The genome analysis of the Chiifu-401-42 line has been completed and published as a reference genome for Chinese cabbage [16] and it is continuously updated. In total, 41,174 protein-coding genes have been identified and about 1,000 genes have been determined to exist only in the Chinese cabbage. Several databases, such as BRAD [17] BrGDB in PlantGDB [18], and Ensembl [19] provide genetic data for different *Brassica* crops.

In the present study, we constructed the pseudomolecule for the inbred line CT001 of the Chinese cabbage to provide a basis for genomic and epigenetic analysis. The constructed pseudomolecule will constitute a basis for genomic and epigenetic analysis of Chinese cabbage, especially in CT001. In addition, spontaneous mutation analysis of Chinese cabbage was carried out to estimate the natural variation derived from sexual propagation.

## Material and methods

### DNA library construction and sequencing

Total genomic DNA of the Chinese cabbage ‘CT001’ was extracted from young leaves of a single plant using sodium dodecyl sulfate lysis buffer with a modified version of the firstly described by Dellaporta et al [20].

DNA libraries were constructed using a TruSeq DNA PCR-Free Kit (Illumina, San Diego, CA, USA) and library quality control (QC) was performed using the Bioanalyzer DNA ChIP (Agilent Technologies, Santa Clara, CA, USA) to produce short single or paired-end reads on Illumina HiSeq 2000 machines (USA). Illumina paired-end sequencing with 150 bp insert size libraries and Illumina mate pair sequencing with large insert (3 and 5 kb) libraries were performed. Data from short-insert paired-end sequencing and those from mate pair sequencing were combined and the gaps between the neighboring scaffolds were filled with 100 Ns. Raw sequences were deposited in NCBI sequence read archive (SRA) with the accession number SRR9190268, belonging to BioProject accession number PRJNA546028.

### Genome assembly and alignment to reference genome

Raw paired-end and mate pair reads were quality trimmed and mapped against the Chiifu reference genome version. 1.5 (available at http://brassicadb.org/brad/). We used the AllPaths-LG assembler from the Broad Institute [21] with default parameters for *de novo* assembly of the two trimmed sequence data. The CT001 genome and Chiifu reference genome were compared using Nucmer [22] and contigs with significant hits were selected at various stages. The assembled contigs have been deposited at NCBI belonging to BioProject accession number PRJNA385249. After the contigs were confirmed, we put 100 Ns between two contigs to represent gaps and complete the pseudomolecule of CT001.

In addition, dot plot comparison of the CT001 pseudomolecule and Chiifu reference genome was carried out. The CT001 assemblies were aligned to the Chiifu genome using Nucmer [22] and the resulting alignment was filtered. Subsequently, dot plotting was performed using MUMmerplot [23] by chromosome.

### RNA extraction and RNA-Seq

For RNA sequencing, seeds of Chinese cabbage CT001 were sown in a greenhouse of Kyung Hee University (Yong In, South Korea). Total RNA was extracted from the young leaves, roots, and apical buds of CT001 using a TaKaRa MiniBEST Plant RNA Extraction Kit (TaKaRa, Otsu, Japan) according to the manufacturer’s instructions.

RNA sequencing libraries were constructed from three samples using a TruSeq Stranded mRNA Sample Preparation Kit (Illumina, USA). The constructed library was quantified using Bioanalyzer DNA Chip (Agilent Technologies, USA) and then sequenced on a HiSeq X (Illumina, USA). In order to improve the accuracy of the results, the adapter/quality trimming was performed using the Trimmomatic program [24]. Trimming conditions were as follows: ILLUMINACLIP:TruSeq3-PE.fa:2:30:10 SLIDINGWINDOW:4:20 LEADING:3 TRAILING:3 MINLEN:50. Subsequently, mapping was performed on the CT001 reference genome based on the trimmed sequence using HiSat2 [25]. We assembled properly paired mapped reads using the StringTie program [26,27]. Transcripts obtained from the three tissues were subjected to assembly merge using TACO [28]. The RNA-seq data are available at the NCBI under accession number SRR9190266, SRR9190267, and SRR9190271.

### Gene annotation

For genome annotation of the *B*. *rapa* ssp. *pekinensis* inbred line CT001, reference-based transcriptome assembly was conducted. In addition to the generated RNA-Seq reads of the three tissues of CT001, we also used six sets of RNA data for Chinese cabbage obtained from the National Center for Biotechnology Information (NCBI). Obtained RNA-Seq data were generated at Kobe University from 10- and 14-day-old leaves and 2- and 6-day-old cotyledons (NCBI SRA accessions: DRX028138, DRX028140, DRX028143, and DRX028147) and at the University of Arizona from unfertilized ovule and 10-day post-fertilization seeds (NCBI SRA accessions: SRX3651784, SRX3651786).

A total of nine RNA-Seq data sets were subjected to reference-guided transcriptome assembly after eliminating adaptor and low quality sequences using Trimmomatic [24]. Mapping of the RNA-Seq reads against the CT001 pseudomolecule was conducted using Hisat2 [25]. The mapped reads from each sample were assembled and the resulting transcriptome was merged using StringTie [26,27]. For annotation purposes, the longest peptide was selected with TransDecoder (https://transdecoder.github.io). Based on these results, a gene model for CT001 was constructed. Gene function was annotated based on protein resources from UniProtKB/Swiss-Prot [29], NCBI, and Araport11 [30]. The protein motif was searched using hmmscan (https://www.ebi.ac.uk/Tools/hmmer/search/hmmscan) based on the Pfam database [31] and gene ontology (GO) annotations were conducted by using Blast2GO against the GO and Kyoto Encyclopedia of Genes and Genomes databases.

### Estimation of the spontaneous mutation rate

For analysis of spontaneous mutations in Chinese cabbage, seeds of the inbred line, developed for 6 years, were sown. The seedling from a seed harvested in 2008 was named ‘4’ and the seedling from a seed harvested in 2014 was named ‘4–1’. The mutation accumulation line was generated from the same individual using the single seed descent (SSD) method. Genomic DNA from each seedling was used for library construction and the libraries were sequenced on Illumina NextSeq 500 sequencing systems (Illumina, USA). The trimmed reads of ‘4’ and ‘4–1’ were aligned to the CT001 reference genome using BWA-MEM (v 0.7.17-r1188) [32] under default parameters, and only uniquely mapped reads were retained. Raw reads have been deposited under NCBI BioProject accession number PRJNA546028.

After mapping, we performed variant calling using the Genome Analysis Toolkit (GATK) [33] in two lines as mentioned above. MarkDuplicates in Picard (v 2.10.6) (http://broadinstitute.github.io/picard/) was used to mark PCR duplicates in BAM files, followed by local realignments around indel regions using IndelRealigner in the GATK package (v 3.8). Raw variants were called from each sample using GATK HaplotypeCaller and gvcf files were combined to vcf using GATK GenotypeGVCFs. Also, the chromosomal locations of the identified base substitutions were analyzed using custom scripts.

For validation of the identified mutations, seeds of inbred line harvested in 2008 and 2014 were germinated and genomic DNA was extracted from leaf tissues of each seedling. In addition, total RNA was extracted from root tissues of each seedling since SNP has occurred at exonic region of the gene that is expected to function as root meristem growth factor. To improve the reliability of the sequence analysis, DNAs and RNAs isolated from two seedlings of ‘4’ line and ten of the ‘4–1’ line were used for PCR and RT-PCR amplification, respectively. PCR analysis was conducted with SNP flanking primer sets (S7 Table) and the PCR products were then eluted using the NucleoSpin Gel and PCR Clean (cat#740609; Macherey-nagel, Düren, Germany). The sequences of the PCR products were obtained from Macrogen® (Macrogen Co., Seoul, Korea) and analyzed. To validate the mutation identified on the exon, named as ‘sm2’, RT-PCR was conducted with primers in exon and 3**′** UTR of the corresponding gene, and the amplicons were eluted and sequenced (S7 Table). The nucleic acid sequences were aligned to confirm the spontaneous mutation. The deduced amino acid sequences were then analyzed to determine if the base substitution causes change in the polypeptide produced.

To calculate the mutation rate, high quality variants were selected by applying a read depth between 10 and 80, bi-allelic sites, and genotype quality threshold (GQ) > 15, 20, 25, and 30 [34]. According to our criteria, mutation sites with the quality cutoff exceeding GQ > 20 were determined and the mutation rate was then calculated with the 95% confidence interval of the Poisson rate. The mutation rate was calculated by dividing the average frequency of mutations per base pair by the number of generations [35]. The spontaneous mutation rate was calculated using the equation *μ* = *m*/*l*/*g*, where *μ* represents the mutation rate, *m* is the number of single base mutations observed, *l* represents the full length of CT001 genome (219,763,438 bp), which were properly mapped to the Chiifu reference genome, and *g* is the total number of generations.

## Results and discussion

### Genome assembly

Data for the CT001 pseudomolecule were generated using paired-end reads with an insert size of 150 bp and mate-paired reads with insert size of 3 kb and 5 kb (S1 Table). Illumina mate paired sequencing generated about 12 million and 53 million reads from the 5 kb and 3 kb insert libraries, respectively. Illumina sequencing produced 32 Gb of sequence data for *B*. *rapa* CT001, representing about 116× genome coverage and included 17 Gb Illumina paired-end reads (61×) and 15 Gb mate-paired reads (55×).

A total of 28,612 contigs were created, containing 231.9 Mb with a minimum length of contigs representing 50% of the assembly (N50) of 13.7 kb. When comparing the CT001 and Chiifu reference (ver. 1.5) sequences, 28,612 contigs were aligned and 25,205 contigs were anchored onto the Chiifu genome (S2 Table). The genome assembly had a contig N50 size of 14.1 kb and the gaps between contigs were filled with 100 Ns; the scaffolds were ordered along the 10 chromosomes of *B*. *rapa* (S2 Table). The genome assembly of the CT001 pseudomolecule eventually included 219.8 Mb of mapped sequences, which covered 85.4% of the Chiifu genome; the coverage rates were similar on each chromosome, ranging from 82.1% to 89.1% (Table 1).

**Table 1:** Chromosomal mapping of *Brassica rapa* inbred line ‘CT001’ contigs to the *B*. *rapa* variety ‘Chiifu’ reference genome.

Chromosome	Length of Chiifu genome (bp)	Contig count of CT001	Covered CT001 sequences (bp)	Average coverage (%)
A01	26,791,028	2,990	21,988,864	82.1
A02	26,939,826	2,849	22,748,939	84.4
A03	31,765,688	2,751	28,297,308	89.1
A04	19,269,589	2,006	16,628,331	86.3
A05	25,303,532	2,370	21,181,456	83.7
A06	25,210,368	2,599	21,417,483	85.0
A07	25,876,096	2,416	22,352,648	86.4
A08	20,826,945	2,047	18,034,844	86.6
A09	38,884,800	3,917	32,748,158	84.2
A10	16,405,180	1,430	14,365,407	87.6
Genome	257,273,052	25,375	219,763,438	85.4

**Fig 1:**
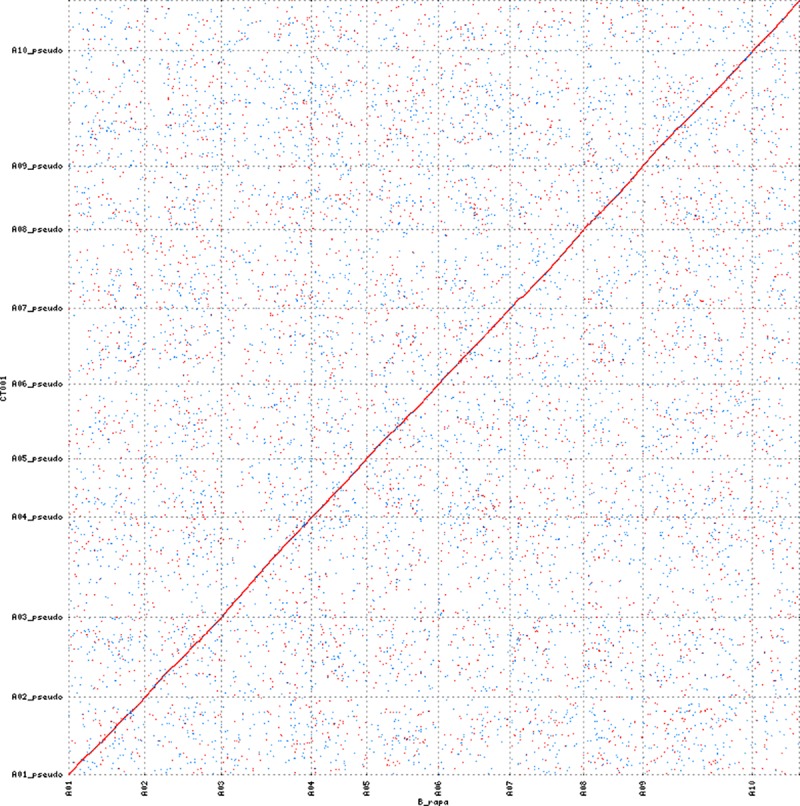
Dot plot comparison between *Brassica rapa* ‘CT001’ and ‘Chiifu’ genome by chromosome. Whole-genome nucleotide sequence alignment of the CT001 scaffolds (plotted on y-axis) against the Chiifu reference sequence ver1.5 (plotted on x-axis). Red and blue lines represent sequences aligned in forward and reverse directions, respectively. The alignment was filtered by percentage identity ≥ 95 and alignment length ≥ 500 bp.

Dot plot comparisons showed that the assembled CT001 strands were significantly aligned against the Chiifu reference genome in all chromosomes (Fig 1). A continuous diagonal line, indicating a relatively high similarity between the CT001 pseudomolecule and the Chiifu reference sequence, was observed. The CT001 pseudomolecule was thus determined to be well constructed along the Chiifu reference genome.

The constructed CT001 pseudomolecule facilitates the understanding of the entire genome sequence of CT001, a Chinese cabbage line used for the traditional or molecular breeding. The obtained pseudomolecule will speed up and increase the accuracy of the analysis of the genetic and epigenetic variations that occur during regeneration and transformation.

### Annotation

RNA sequencing of CT001 was performed using three different tissues: leaf, root, and apical bud. In total, 5.4 Gb of RNA-Seq data was generated from young leaves of CT001, 5.1 Gb from the root, and 4.7 Gb from the apical bud using the Illumina HiSeq platform. After trimming, the transcripts were reduced to 3.5 Gb, 3.2 Gb, and 3.0 Gb per tissue sample, respectively (S3 Table and S4 Table). To improve the accuracy of the analysis, a reference-guided transcriptome assembly was performed with a total of nine transcriptome data, including additional RNA-Seq data from NCBI, and the assembled transcriptome data were compared with the reference protein data that were successfully mapped to the CT001 pseudomolecule.

Based on the analyzed transcripts, 38,567 gene models were determined. The final summary of the annotation is shown in Table 2. Of these, 34,310 transcripts were already annotated on the Chiifu reference genome and over 99% of the length of each transcript was mapped onto the CT001 pseudomolecule and 4,257 transcripts remained unannotated. These transcripts were considered incompletely annotated in Chiifu genome ver 1.5. Additionally, sequences of the unannotated transcripts were aligned to Chiifu reference with a BLAST e-value <10^−6^. Among the 4,257 transcripts, sequences of 4,201 transcripts were found to be present on the Chiifu genome sequence but seemed to be unannotated owing to the parameters for gene annotation in the reference genome. However, 56 transcripts were not aligned to the Chiifu genome. The majority of these were considered genes of unknown function (S5 Table). Thirteen transcripts were annotated in *Brassica* crops including *B*. *oleracea* and *B*. *napus*. However, only one was functionally identified, as a magnesium transporter, and 12 transcripts were not characterized. The subset of 43 unannotated transcripts did not yield a BLAST alignment and the functions of the genes are unknown. Further research for these transcripts is required to prove that these transcripts are unique to CT001 or not identified yet in the Chiifu genome.

**Table 2:** Gene annotation of *Brassica rapa* ‘CT001’.

Feature	Value
Number of gene models	38,567
Total gene length (bp)	72,036,748
Number of single exon genes	9,758
Number of multiple exon genes	28,809
Average gene length (bp)	1,868
Total number of exon	185,524
Average number of exon/gene	4.8
Average exon length (bp)	231.7
Total number of intron	146,967
Average number of intron/gene	3.8
Average intron length (bp)	182.8
Number of genes with known plant protein homology (e-value <10^−6^)	35,837

**Fig 2:**
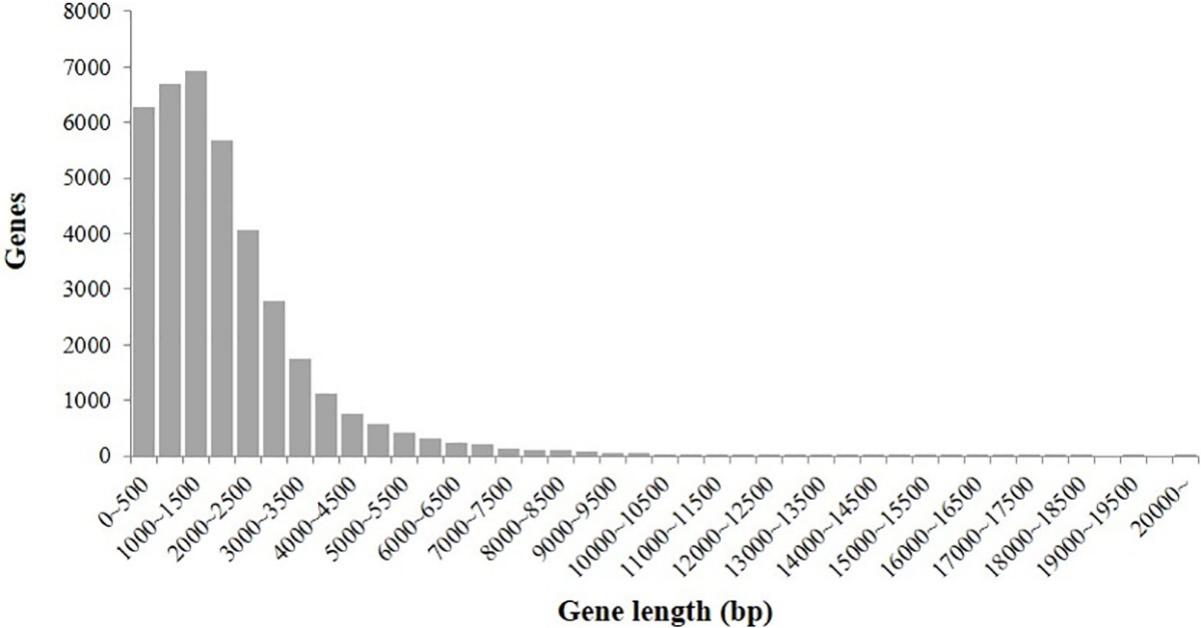
Distribution of gene length in the *Brassica rapa* ‘CT001’ pseudomolecule. The y-axis represents the number of genes with a certain length. Genes range in size from 0.1 kb to 39.2 kb.

Final assembled transcripts were annotated in CT001 with a mean of 4.8 exons per gene, which is less than that in *Arabidopsis* (5.41) but higher than that in other plant species. For example, the number of exons per gene was 3.8 in rice [36], 4.1 in maize [37], 4.3 in sorghum [38], and 4.5 in *B*. *oleracea* [39]. The total length of the gene was 72,036,748 bp, the average gene length was 1,868 bp, and the median was 1,451 bp (Fig 2). The number of transcripts was 47,909, and the total length of the transcripts was 112,907,435 bp. The number of exons was 185,524 with an average length of 231.7 bp. The average exon size of the CT001 pseudomolecule was considerably shorter than that of *Arabidopsis* (258.3 ± 11.9 bp) but longer than that of *B*. *oleracea* (211.2 ± 8.6 bp) [39]. The average intron length for CT001 pseudomolecule was 182.8 bp. Of the 38,567 gene models, 35,837 (93%) genes had a known protein homology.

### Detection of spontaneous mutations

As a general model for research on unexpected genomic mutations, such as somaclonal variations in regenerated and transgenic plants, the spontaneous mutation rate should be calculated. [40,41,42]. To estimate the rate of spontaneous mutations in sexually propagated CT001, two individual plants six generations apart were sequenced to a coverage depth of between ~18× and ~21× per individual. About 5 Gb and 6 Gb of trimmed data were mapped to the CT001 pseudomolecule and 34 million and 41 million cleaned reads were obtained in ‘4’ and ‘4–1’, respectively (S6 Table and S7 Table).

**Fig 3:**
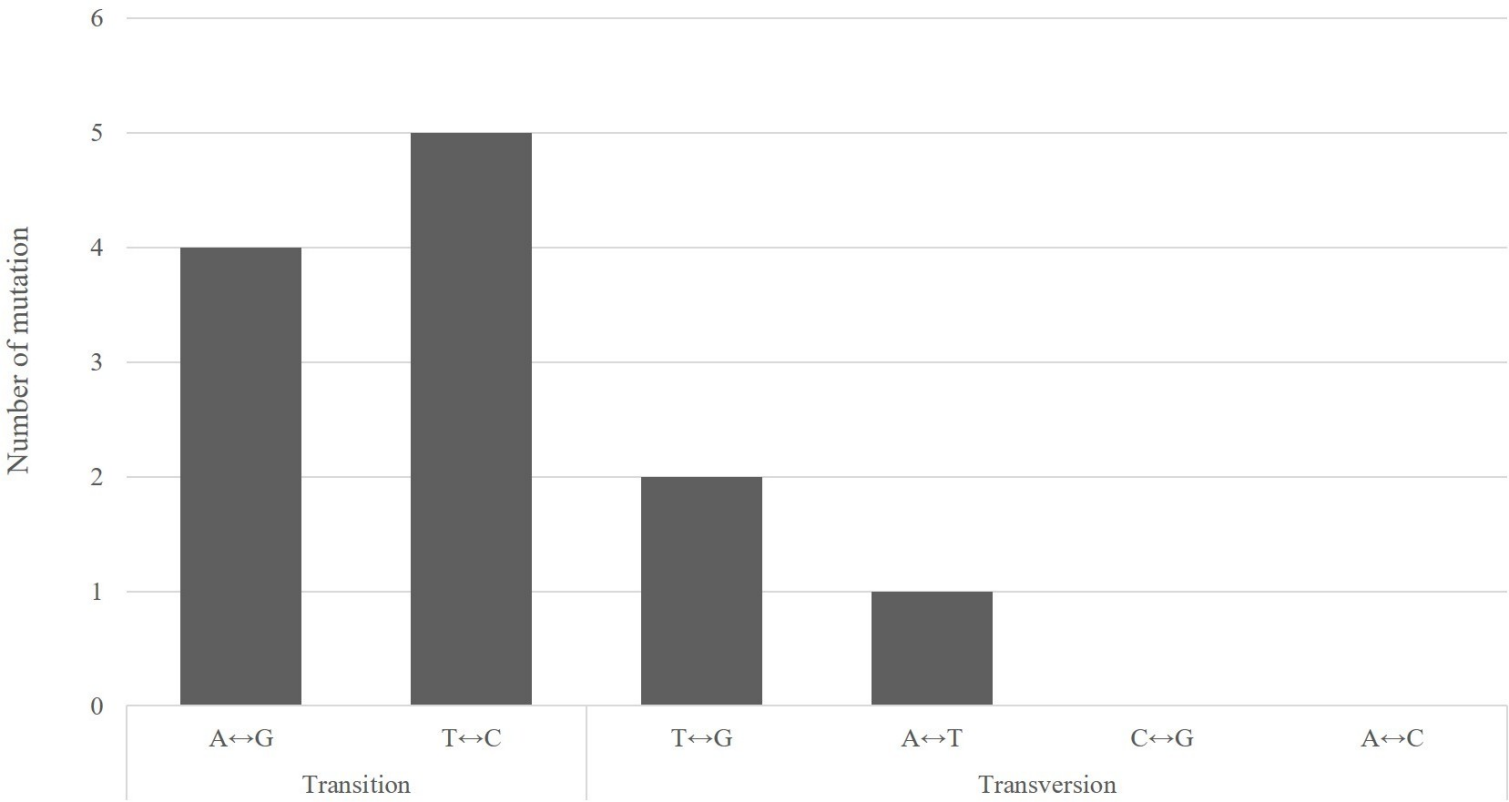
Proportions of base substitution types of the 12 identified mutation. Transitions made up the majority of the substitutions.

In the combined data for the two individual plants, 12 base substitutions were identified (Table 3). Of these base substitutions, 9 were transitions (A/G, T/C) and 3 were transversions (T/G, A/T, A/C, C/G) (Fig 3). In particular, spontaneous mutations were mainly transitions, either A/G and T/C. The sites of spontaneous mutations in CT001 are summarized in Table 3. Most mutations occurred in the intergenic region, while a few were found in the coding sequence (Table 4).

**Table 3:** Number of spontaneous mutations and their distribution in the *Brassica rapa* ‘CT001’ genome.

Features	Transition	Transversion
Exon	1	0
Intron	1	2
Intergenic	7	1
Total mutation	9	3

**Table 4:** Distribution of mutations across the *Brassica rapa* ‘CT001’ chromosomes and related gene information.

	Chromosome	Location	Ref	Alt	SNP	Type*	Gene ID	Gene description
sm1	A01_pseudo	1447959	C	T	Ts	Intergenic		
sm2	A01_pseudo	8787755	A	G	Ts	Exon	CT001_A01017300	PREDICTED: Root meristem growth factor 6
sm3	A01_pseudo	10776639	A	G	Ts	Intergenic		
sm4	A02_pseudo	16640494	C	T	Ts	Intergenic		
sm5	A02_pseudo	18875414	A	G	Ts	Intron	CT001_A02067580	PREDICTED: FACT complex subunit SSRP1
sm6	A03_pseudo	14956214	T	G	Tv	Intron	CT001_A03107270	PREDICTED: B3 domain-containing protein REM8-like isoform X2
sm7	A04_pseudo	10593814	C	T	Ts	Intergenic		
sm8	A06_pseudo	5646555	T	G	Tv	Intron	CT001_A06206720	PREDICTED: receptor like protein 30-like
sm9	A07_pseudo	16467555	A	T	Tv	Intergenic		
sm10	A09_pseudo	5066860	A	G	Ts	Intergenic		
sm11	A09_pseudo	10751579	C	T	Ts	Intergenic		
sm12	A09_pseudo	12032695	C	T	Ts	Intergenic		

Notes

* Annotation of single nucleotide polymorphism (SNP) regions

**Abbreviations:** Ref, reference; Alt, altered; Ts, transition; Tv, transversion.

The mutation rate was estimated at 9.10 × 10^−9^ base substitutions per site per generation (95% confidence interval: 3.95 × 10^−9^–1.42 × 10^−8^), which is about 1.3-fold higher than the mutation rate in *Arabidopsis* (7 × 10^−9^ bp/site/generation) [43] but approximately 10-fold lower than in rice (10^−7^–10^−8^ bp/site/generation) [44]. Moreover, the mutation rate was expected to be higher in plants that have undergone regeneration or transformation than in sexually propagated plants.

In this study, to compensate the limitations of the n = 2 mutation analysis, we provided molecular evidence independent of the resequencing analysis for the mutations. To confirm the 12 spontaneous mutations identified from resequencing data analysis, PCR analysis was conducted (S1 Fig). The amplicons were produced as expected sizes (S7 Table), and the amplified products were eluted and sequenced. The alignments of nucleic acid sequences from amplicons showed that the spontaneous mutations occurred in 4–1, as analyzed by comparing the resequencing data of 4 and 4–1 (S2 Fig).

For the mutation (sm2) identified on the exon, RT-PCR analysis and sequencing of the eluted RT-PCR amplicons were also carried out. The amplicons with expected sizes were produced (S3A Fig) and the sequencing result showed that spontaneous mutation occurred on the exonic region in 4–1, (right before the stop codon) (S3B Fig). However, the silent mutation has identified as the codon has changed TCA into TCG, but both are redundant codons for serine. In conclusion, the spontaneous mutation in exonic region did not alter the polypeptide sequence.

## Conclusion

In this study, we have performed full genome sequencing by using NGS technologies and reference guided assembly on the *Brassica rapa* ssp. *pekinensis* inbred line CT001. Over 85% of the reference genome was covered by the assembled sequences. For gene prediction, the constructed pseudomolecule was annotated with a number of transcripts of *B*. *rapa* inbred lines. Combined with these data, we present the draft genome of *B*. *rapa* inbred line CT001 developed using a reference-guided assembly strategy. In addition, the spontaneous mutation rate in CT001 was analyzed to estimate the variations induced by sexual propagation. The identified mutations were validated by PCR, RT-PCR and sequence analysis. The mutation rate (9.10 × 10^−9^ base substitutions per site per generation) in CT001 was slightly higher than that in *Arabidopsis*, but much lower than that in rice. The created pseudomolecule and determination of spontaneous mutation of CT001 in this study is expected to contribute to a better understanding of the *B*. *rapa* genome and to be utilized in future genomic studies.

## Supporting information

S1 Table Raw and trimmed data for paired-end reads and mate-paired reads. (PDF)

S2 Table Sequencing and genome assembly statistics for the CT001 pseudomolecule. (PDF)

S3 Table Raw and trimmed transcriptome data for three tissues from CT001. (PDF)

S4 Table Mapping of transcriptome data for three tissues from CT001. (PDF)

S5 Table Raw and trimmed data for spontaneous mutation in CT001. (PDF)

S6 Table Mapping of data for spontaneous mutation in CT001. (PDF)

S7 Table Primers for PCR confirmation of the identified spontaneous mutation. (PDF)

S1 Fig PCR confirmation of the identified spontaneous mutations. **Sm1~sm12** represent the target mutation described in S7 Table. Each lane represents the analyzed ‘4’ and ‘4–1’ lines. PCR amplification was performed using the DNA isolated from two seedlings of ‘4’ line and ten of the ‘4–1’ line to improve the reliability of the sequence analysis.
(PDF)

S2 Fig Sequence analysis for the identified spontaneous mutations. Sequences of two seedlings of ‘4’ line, as control, and ten of the ‘4–1’ line were compared and showed that mutations have occurred as analyzed by comparing the resequencing data. The red boxes indicate the sequences of the target mutation locus.
(PDF)

S3 Fig RT-PCR and cDNA sequence analysis of the mutation located in exonic region. (A) RT-PCR confirmation for the sm2 mutation. (B) cDNA sequence analysis of the sm2 mutation occurred in exon. The spontaneous mutation was validated in exonic region but it did not alter the polypeptide sequence as both codons, TCA and TCG, encode serine. CDS, coding sequence; UTR, untranslated regions.
(PDF)
