# Supplementary material for: Construction of pseudomolecule sequences of Brassica rapa ssp. pekinensis inbred line CT001 and analysis of spontaneous mutations derived via sexual propagation
Source: PLoS One. 2019 Sep 9;14(9):e0222283. doi: 10.1371/journal.pone.0222283 (PMC6733507; doi:10.1371/journal.pone.0222283)
Supplement: S1 Table — (PDF) [file pone.0222283.s001.pdf]

S1 Table. Raw and trimmed data for paired-end reads and mate-paired reads

	No. of raw reads	raw length (bp)	No. of trimmed reads	Trimmed length (bp)	No. of Duplication removed reads	Duplication removed length (bp)
Paired-end reads	174,412,558	26,229,517,161	133,576,982	16,992,825,814	-	-
CT001-5kb	71,022,342	10,573,040,151	55,682,824	7,373,427,633	11,980,350	599,017,500
CT001-3kb	69,896,210	10,457,196,149	58,297,272	7,975,806,377	52,582,762	2,629,138,100
