# Supplementary material for: Construction of pseudomolecule sequences of Brassica rapa ssp. pekinensis inbred line CT001 and analysis of spontaneous mutations derived via sexual propagation
Source: PLoS One. 2019 Sep 9;14(9):e0222283. doi: 10.1371/journal.pone.0222283 (PMC6733507; doi:10.1371/journal.pone.0222283)
Supplement: S2 Table — (PDF) [file pone.0222283.s002.pdf]

S2 Table. Sequencing and genome assembly statistics for the CT001 pseudomolecule.

	Total count	Total length (bp)	N50	L50	Maximum length (bp)	Average Length (bp)
Assembled contig (>=1kb)	28,794	232,163,471	13,636	5,014	135,534	8,063
Chromosome anchored contig	25,375	217,226,938	14,026	4,580	135,534	8,560
Unanchored contig	3,419	14,936,533	8,708	500	79,836	4,369
Final scaffold [a]	10	219,763,438	*	*	*	*

[a] Scaffolds were made using ordered/oriented contigs with 100 Ns
