# Supplementary material for: Construction of pseudomolecule sequences of Brassica rapa ssp. pekinensis inbred line CT001 and analysis of spontaneous mutations derived via sexual propagation
Source: PLoS One. 2019 Sep 9;14(9):e0222283. doi: 10.1371/journal.pone.0222283 (PMC6733507; doi:10.1371/journal.pone.0222283)
Supplement: S3 Table — (PDF) [file pone.0222283.s003.pdf]

S3 Table. Raw and trimmed transcriptome data for three tissues from CT001

	Raw reads	Raw bases (bp)	Trimmed reads	Trimmed bases (bp)
CT001_leaf	35,889,934	5,419,380,034	23,783,936	3,501,295,981
CT001_root	33,663,548	5,083,195,748	21,962,299	3,229,212,512
CT001_apical bud	31,437,162	4,747,011,462	20,140,662	2,966,503,492
