# Supplementary material for: Construction of pseudomolecule sequences of Brassica rapa ssp. pekinensis inbred line CT001 and analysis of spontaneous mutations derived via sexual propagation
Source: PLoS One. 2019 Sep 9;14(9):e0222283. doi: 10.1371/journal.pone.0222283 (PMC6733507; doi:10.1371/journal.pone.0222283)
Supplement: S4 Table — (PDF) [file pone.0222283.s004.pdf]

S4 Table. Mapping of transcriptome data for three tissues from CT001

	Mapped read	Mapped bases (bp)	Unmapped read	Properly paired mapped read	Mapping Coverage
CT001_leaf	22,429,800	3,296,970,550	1,354,136	21,494,322	95.8%
CT001_root	18,717,866	2,753,902,953	1,422,796	17,869,788	95.5%
CT001_ apical bud	21,392,549	3,146,947,783	1,367,547	20,536,510	96.0%
