# Supplementary material for: Construction of pseudomolecule sequences of Brassica rapa ssp. pekinensis inbred line CT001 and analysis of spontaneous mutations derived via sexual propagation
Source: PLoS One. 2019 Sep 9;14(9):e0222283. doi: 10.1371/journal.pone.0222283 (PMC6733507; doi:10.1371/journal.pone.0222283)
Supplement: S5 Table — (PDF) [file pone.0222283.s005.pdf]

S5 Table. Raw and trimmed data for spontaneous mutation in CT001

Name	Raw reads	Raw bases (bp)	Trimmed reads	Trimmed bases (bp)
4	38,643,892	5801,323,367	34,997,560	5,033,120,157
4-1	45,200,982	6,786,926,130	41,032,274	5,907,922,331
