# Supplementary material for: Construction of pseudomolecule sequences of Brassica rapa ssp. pekinensis inbred line CT001 and analysis of spontaneous mutations derived via sexual propagation
Source: PLoS One. 2019 Sep 9;14(9):e0222283. doi: 10.1371/journal.pone.0222283 (PMC6733507; doi:10.1371/journal.pone.0222283)
Supplement: S6 Table — (PDF) [file pone.0222283.s006.pdf]

S6 Table. Mapping of data for spontaneous mutation in CT001

Name	Cleaned read	Properly paired mapped read	Multiple Hit	Unique Hit paired	AVG Depth
4	34,997,560	23,363,692	9,346,482	14,017,210	9.72
4-1	41,032,274	26,311,946	10,530,208	15,781,738	10.87

.
