# Supplementary material for: Construction of pseudomolecule sequences of Brassica rapa ssp. pekinensis inbred line CT001 and analysis of spontaneous mutations derived via sexual propagation
Source: PLoS One. 2019 Sep 9;14(9):e0222283. doi: 10.1371/journal.pone.0222283 (PMC6733507; doi:10.1371/journal.pone.0222283)
Supplement: S7 Table — (PDF) [file pone.0222283.s007.pdf]

S7 Table. Primers for PCR confirmation of the identified spontaneous mutation.

Target mutation	Primer name	Nucleotide sequences (5′ to 3′)	Product size
sm1	sm1 F	TGGAGGGTTGGAGACTCTC	259 bp
	sm1 R	CGATCTCTACAATGATAGGTAG	
sm2	sm2 F	GTCGTTTAGGGCTTCACAAG	226 bp
	sm2 R	GCATATAACAGTGTGGTGATTC	
sm2-exon	sm2-exon F	GTCGTTTAGGGCTTCACAAG	190 bp
	sm2-UTR R	GAGTAATTTAAGCCTTCTCGTTG	
sm3	sm3 F	CGAGATGTACCGAGTGAGG	300 bp
	sm3 R	GGCAAGTTAGGCGTGTTGG	
sm4	sm4 F	TAAGGAACATCAACCGTGATC	338 bp
	sm4 R	CGATAGAACCCGAAACATTCA	
sm5	sm5 F	GGTGAGGTGGACTTGAATGG	279 bp
	sm5 R	CTTGAAGCTGAGTCTGTGAC	
sm6	sm6 F	GTTTAATGTTTCCTCGGACTC	170 bp
	sm6 R	GGCTTTCACATAATGCTTCTC	
sm7	sm7 F	GGGTGTGAACTATTTACTTG	298 bp
	sm7 R	GCAAATCATATCTTCCTAAC	
sm8	sm8 F	CGAGGACAATCTTGGACTC	242 bp
	sm8 R	GGTTGCGACGAAACTTCTAC	
sm9	sm9 F	CCAGTACCATATCTTACGAG	314 bp
	sm9 R	TAGGTGGTAGGTTATTATGG	
sm10	sm10 F	GCAGGGTTGGTTGAGATAT	224 bp
	sm10 R	GAATGATAGACGGACCACTC	
sm11	sm11 F	GGACGATCAGGGAGTACAAG	363 bp
	sm11 R	GACTTGTTTACTGACCCTGC	
sm12	sm12 F	TGACACCCATACTATGACTTTG	213 bp
	sm12 R	CCTAGCATTGGGTTATTGTG	
