# Supplementary material for: Construction of pseudomolecule sequences of Brassica rapa ssp. pekinensis inbred line CT001 and analysis of spontaneous mutations derived via sexual propagation
Source: PLoS One. 2019 Sep 9;14(9):e0222283. doi: 10.1371/journal.pone.0222283 (PMC6733507; doi:10.1371/journal.pone.0222283)
Supplement: S1 Fig — Sm1~sm12 represent the target mutation described in S7 Table. Each lane represents the analyzed ‘4’ and ‘4–1’ lines. PCR amplification was performed using the DNA isolated from two seedlings of ‘4’ line and ten of the ‘4–1’ line to improve the reliability of the sequence analysis. (PDF) [file pone.0222283.s008.pdf]

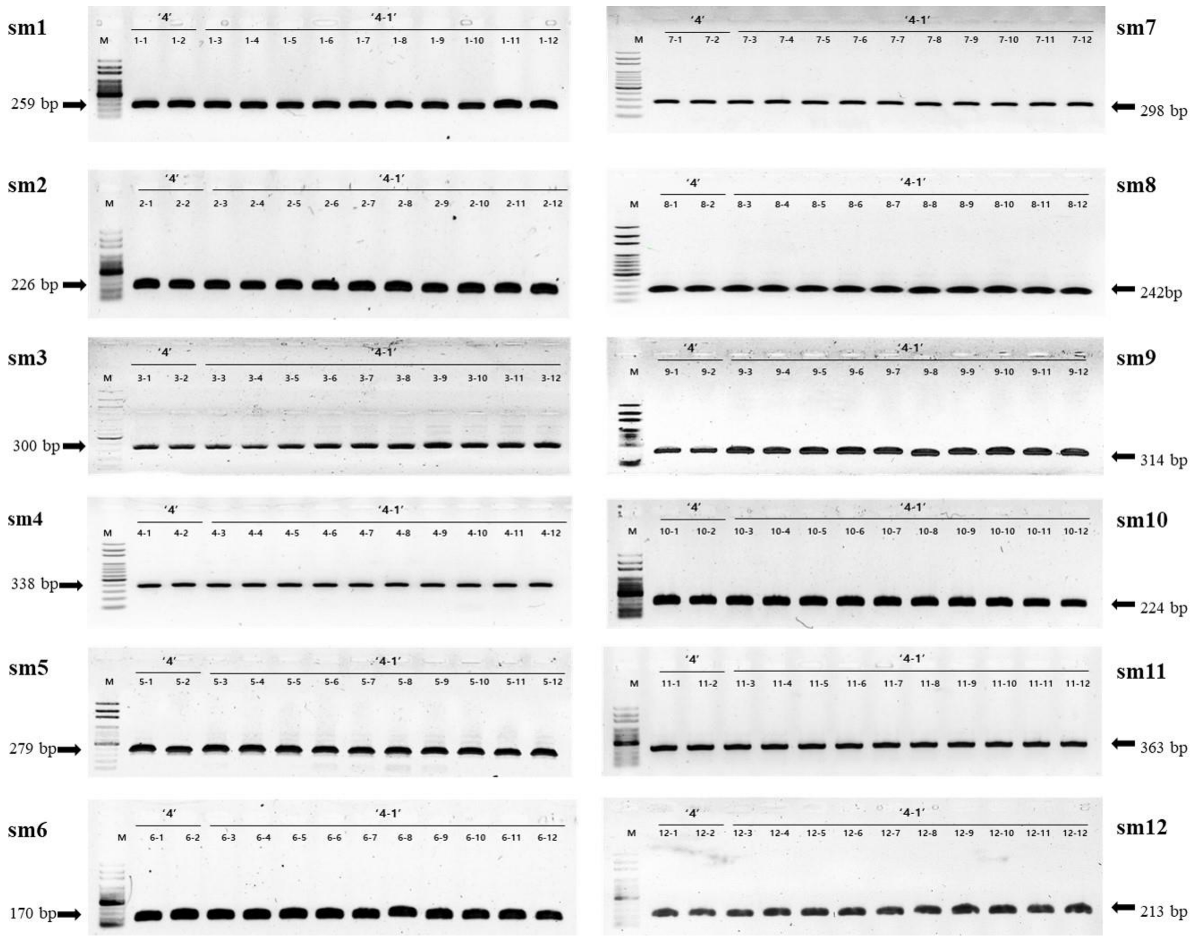

S1 Fig. PCR confirmation of the identified spontaneous mutations. Sm1~sm12 represent the target mutation described in S7 Table. Each lane represents the analyzed ‘4’ and ‘4-1’ lines. PCR amplification was performed using the DNA isolated from two seedlings of ‘4’ line and ten of the ‘4-1’ line to improve the reliability of the sequence analysis.
