# Supplementary material for: Construction of pseudomolecule sequences of Brassica rapa ssp. pekinensis inbred line CT001 and analysis of spontaneous mutations derived via sexual propagation
Source: PLoS One. 2019 Sep 9;14(9):e0222283. doi: 10.1371/journal.pone.0222283 (PMC6733507; doi:10.1371/journal.pone.0222283)
Supplement: S2 Fig — Sequences of two seedlings of ‘4’ line, as control, and ten of the ‘4–1’ line were compared and showed that mutations have occurred as analyzed by comparing the resequencing data. The red boxes indicate the sequences of the target mutation locus. (PDF) [file pone.0222283.s009.pdf]

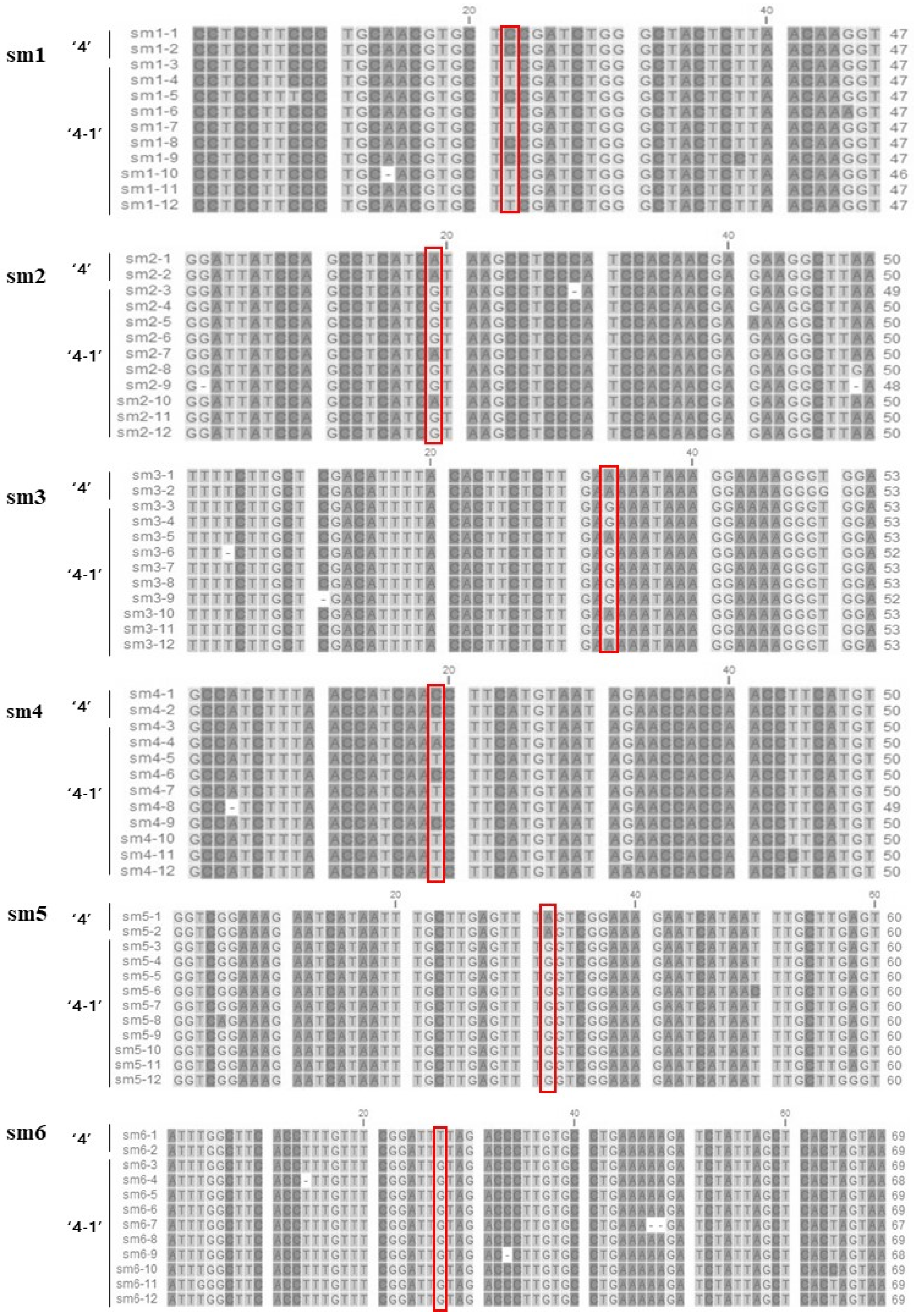

S2 Fig. Sequence analysis for the identified spontaneous mutations. Sequences of two seedlings of ‘4’ line, as control, and ten of the ‘4-1’ line were compared and showed that mutations have occurred as analyzed by comparing the resequencing data. The red boxes indicate the sequences of the target mutation locus.

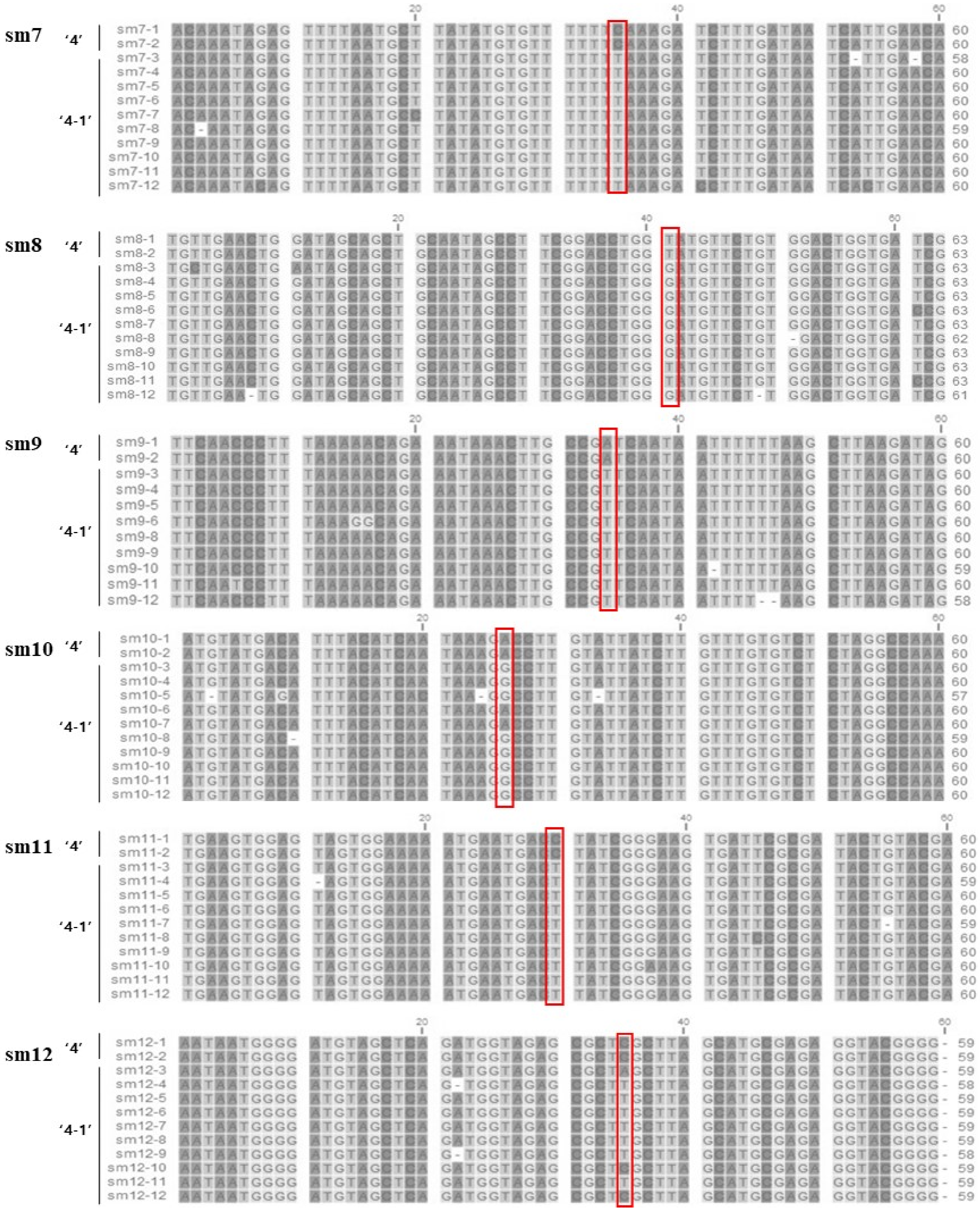

S2 Fig. Sequence analysis for the identified spontaneous mutations (continued).
