# Supplementary material for: Construction of pseudomolecule sequences of Brassica rapa ssp. pekinensis inbred line CT001 and analysis of spontaneous mutations derived via sexual propagation
Source: PLoS One. 2019 Sep 9;14(9):e0222283. doi: 10.1371/journal.pone.0222283 (PMC6733507; doi:10.1371/journal.pone.0222283)
Supplement: S3 Fig — (A) RT-PCR confirmation for the sm2 mutation. (B) cDNA sequence analysis of the sm2 mutation occurred in exon. The spontaneous mutation was validated in exonic region but it did not alter the polypeptide sequence as both codons, TCA and TCG, encode serine. CDS, coding sequence; UTR, untranslated regions. (PDF) [file pone.0222283.s010.pdf]

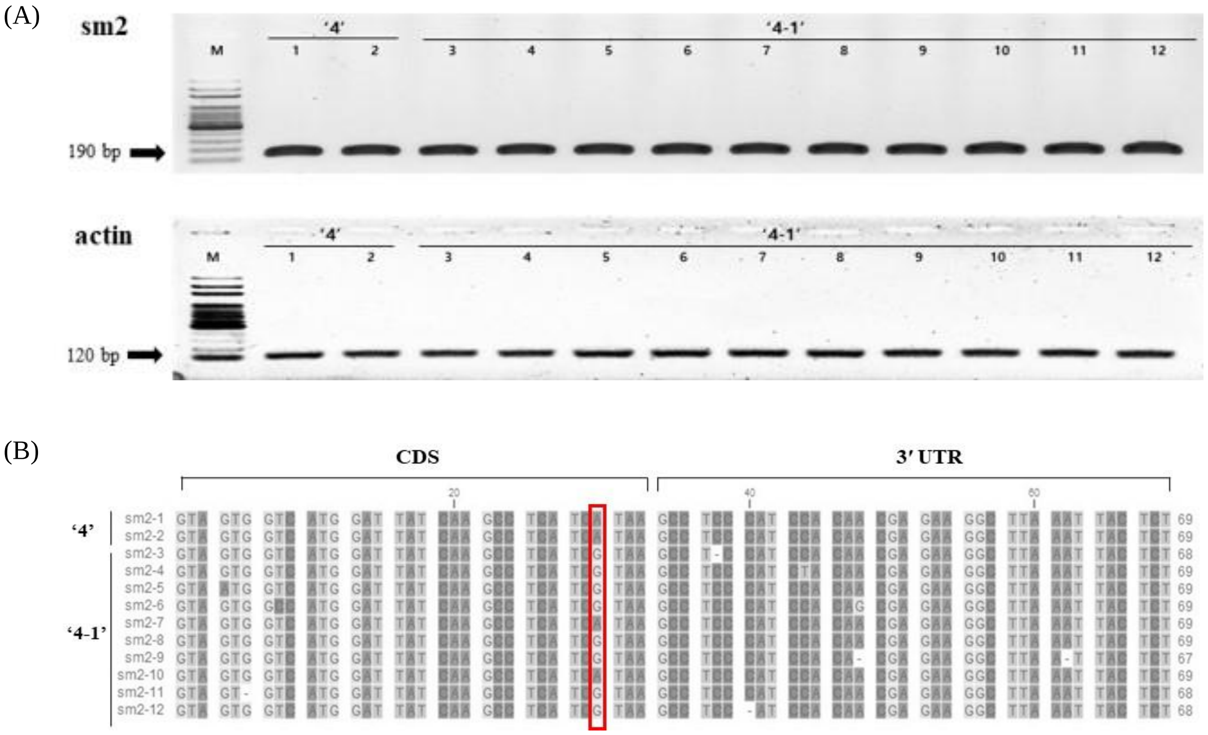

S3 Fig. RT-PCR and cDNA sequence analysis of the mutation located in exonic region. (A) RT-PCR confirmation for the sm2 mutation. (B) cDNA sequence analysis of the sm2 mutation occurred in exon. The spontaneous mutation was validated in exonic region but it did not alter the polypeptide sequence as both codons, TCA and TCG, encode serine. CDS, coding sequence; UTR, untranslated regions.
